# Functionalization Routes for Keratin from Poultry Industry Side-Streams—Towards Bio-Based Absorbent Polymers

**DOI:** 10.3390/polym15020351

**Published:** 2023-01-09

**Authors:** Victor M. Perez-Puyana, Antonio J. Capezza, William R. Newson, Carlos Bengoechea, Eva Johansson, Antonio Guerrero, Mikael S. Hendeqvist

**Affiliations:** 1 Departamento de Ingeniería Química, Facultad de Química, Escuela Politécnica Superior, Universidad de Sevilla, 41012 Sevilla, Spain; 2 Fibre and Polymer Technology Department, KTH Royal Institute of Technology, 11428 Stockholm, Sweden; 3 Department of Plant Breeding, Swedish University of Agricultural Sciences, Box 190, SE-23422 Lomma, Sweden

**Keywords:** poultry feathers, biopolymer, functionalization, succinic anhydride, EDTAD, superabsorbent

## Abstract

Keratin is a largely available protein that can be obtained from the ca. 3 million tons of feathers that the European poultry industry produces as a side-stream. Here, the functionalization of keratin from poultry feathers was evaluated using a one- versus two-stage process using two functionalization agents (succinic anhydride-SA and ethylene dianhydride-EDTAD). The functionalization resulted in the keratin having improved liquid swelling capacities, reaching up to 400%, 300%, and 85% increase in water, saline, and blood, respectively, compared to non-functionalized keratin. The highest swelling was obtained for samples functionalized with EDTAD (one-stage process), while the highest saline uptake was noted for samples processed with 25 wt% SA (two-stage process). Swelling kinetics modeling indicated that the water uptake by the functionalized samples takes place in two steps, and the EDTAD samples showed the highest diffusivity. It is demonstrated that the one-stage functionalization of keratin utilizing EDTAD results in better performance than two-stages, which allows for resource-saving and, thereby, protecting the environment. The results show some potential for the keratin to be utilized as liquid absorbent materials in water, saline, and blood uptake applications. Using keratin from side-streams is an advantage from a sustainability perspective over biomacromolecules that need to be extracted from virgin biomass.

## 1. Introduction

The poultry industry is an important contributor to the total meat production in Europe and other regions worldwide [1,2]. Spain is a leading European meat producer, with chicken meat representing 11.8% of the entire local production [3]. In fact, the preference for chicken as the source of protein has grown by 70% over the last three decades, and poultry livestock are promoted as a food source to meet the protein requirements of human nutrition [4]. The poultry industry generates various types of waste, including feathers, blood, bones, and skin; feather alone represents 5 million tons of waste per year (worldwide) [5]. Its chemical composition mainly consists of carbon (around 35%); small amounts of phosphorous and nitrogen (around 4%); and trace amounts of several elements such as calcium, sodium, manganese, potassium, etc. (around 1–2%) [6].

Keratin protein is the main component in poultry feathers and is also present in hair, nails, skin, and wool [7]. Keratin is a fibrillar protein consisting of ca. twenty amino acids with a high sulfur content (4.0–5.5%) [8]. Due to the high sulfur content of the protein, intra- and intermolecular bonds are formed in and between fibrillar chains, leading to difficulties in solubilizing the protein. Due to its high protein content and hydrophilic character, keratin has been beneficial in several applications, e.g., developing nanoparticles for encapsulation and drug release, hydrogels, scaffolds or tissues for cell regeneration, or in therapeutic applications [9,10,11].

Biorefining waste to extract keratin-based substances from biomass (i.e., proteins, amino acids, hydrolysates, and keratinases) is increasing attention for upcycling and manufacturing sustainable products for various industrial applications. Recently reported applications for the upcycled extracted keratin from waste ranges from biohybrid composites to electrospun fibers [2,12].

Absorbent materials are used in many products/applications where water holding might be important, ranging from diapers and sanitary pads to agriculture and horticulture [13,14]. Absorbent materials are generally derived from non-biodegradable fossil-based acrylic monomers [15]. Recent studies have shown that biopolymers, such as proteins from industrial by-products, can produce sustainable absorbents with similar swelling behavior to their synthetic counterparts while providing biodegradability and low toxicity [16,17,18,19]. Additionally, using keratin from side-streams is an advantage from a sustainability perspective over biomacromolecules that need to be extracted from virgin biomass [20].

Raw materials that can be developed into superabsorbent polymers must be hydrophilic with adequate water absorption capacity [21,22,23]. Some raw materials, e.g., proteins, may require chemical modifications to obtain such characteristics. Functionalization of proteins has been demonstrated to contribute to improved absorption capacity by adding chemically active groups [24,25]. Acylation of the amino acid residues with anhydrides is one of the most commonly utilized functionalization strategies, where succinic anhydride (SA) and ethylenediaminetetraacetic dianhydride (EDTAD) are the most frequently used anhydrides [26,27].

The present study aims to evaluate different functionalization methodologies on keratin extracted from poultry feathers to increase the material’s absorption capacity. The main novelty of this study is the functionalization of keratin using SA and EDTAD in a two-stage (i.e., alkaline extraction followed by functionalization) or a single-stage process (i.e., alkaline extraction and functionalization carried out simultaneously), not reported before in the literature. The liquid swelling and swelling kinetics of the materials obtained were evaluated as related to their potential to be used as absorbents. The results show that the extracted keratin is a promising candidate for future studies aiming at replacing current petroleum-based absorbents used in many single-use articles, producing large amounts of non-biodegradable waste into the environment.

## 2. Materials and Methods

### 2.1. Materials

Poultry feathers were supplied by Paviso Alimentación (Sevilla, Spain). Succinic anhydride (SA) and ethylenediaminetetraacetic dianhydride (EDTAD), used as functionalization reagents, were purchased from Sigma Aldrich (St. Louis, MO, USA). Other reagents, i.e., hydrochloric acid (HCl) or sodium hydroxide (NaOH), were purchased from Panreac Química, S.A. (Barcelona, Spain). All the reagents were of analytical grade and used without purification. Defibronated sheep blood was purchased from Håtunalab AB (Bro, Sweden).

### 2.2. Extraction and Functionalization of Keratin

**Figure 1:**
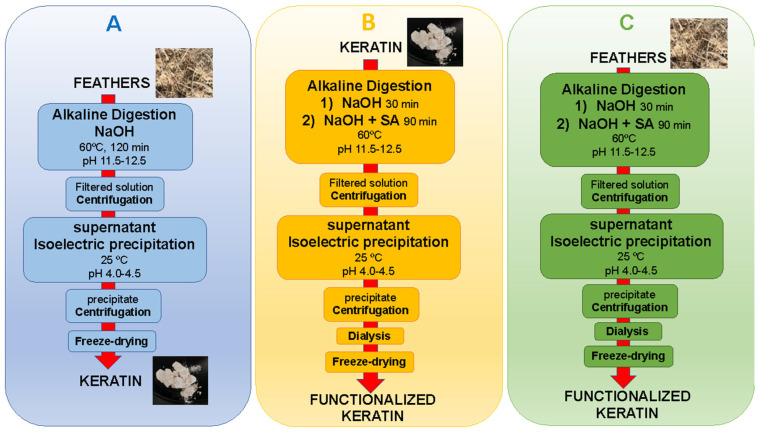
Schematic overview of the two-stage process with Extraction (**A**) and Functionalization (**B**) as separate steps and the one-stage process with Extraction and Functionalization in 1 stage (**C**).

The feathers were washed with soap and water, then rinsed extensively with water, and dried in a conventional oven at 40 °C for 24–48 h. Samples were prepared as described in Table 1, utilizing a two- or one-stage process (as described below). SA or EDTAD were used as functionalization agents at 10 or 25 wt% (relative to the protein content) based on results from previous studies [26,27]. In Table 1, the first number in the sample names refers to the stages of the process (2 or 1), “F” refers to functionalized samples, and “S” or “ED” to the functionalization agent used (S = SA, ED = EDTAD). The last two numbers in the sample names (10 or 25) represent the concentration of functionalization agent used (10% or 25 wt%). As-extracted keratin (KE) was used as a control sample. A schematic overview of the different extraction processes is shown in Figure 1.

The two-stage process started with an extraction step (Figure 1A) and a functionalization step (Figure 1B). To extract the keratin, 40 g of feathers were weighed and dispersed in 200 mL of 2 M NaOH solution. The suspension was kept under magnetic stirring at 500 rpm for 2 h at 60 °C, at a pH of 11.5–12.5. The resulting solution was centrifuged (10,000 rpm for 7 min), the supernatant collected, and then the pH of the supernatant was lowered to the isoelectric point of keratin (pH 4–4.5). After that, the sample was centrifuged (10,000 rpm for 7 min), and the resulting solid was collected, rinsed with water, and lyophilized for 24–48 h to remove all excess water. To functionalize the obtained keratin, 14 g was dispersed in 200 mL of 2M NaOH solution, and the suspension was kept under stirring at 500 rpm at 60 °C. After 30 min stirring, SA was added to the solution, which was kept under agitation for another 1.5 h, maintaining the pH of the solution at 11.5–12.5 with 2 M NaOH. Then, the resulting solution was centrifuged, the supernatant collected, and the supernatant’s pH was decreased to pH 4–4.5. Thereafter, the samples were purified through dialysis utilizing cellulose membranes of 14 kDa cut-off (Ref. D9652 Sigma-Aldrich, St. Louis, MO, USA) and subsequently submerged in distilled water with constant renewal of the distilled water for 72 h. Finally, the samples were frozen at −40 °C for 2 h and freeze-dried for 48 h.

In the one-stage process (Figure 1C), 40 g of feathers were dispersed in 200 mL of 2 M NaOH solution, and the suspension was then stirred at 500 rpm and 60 °C for 30 min. After that, an adequate amount of either SA or EDTAD was added to the solution. The agitation of the solution was kept for another 1.5 h while the pH was maintained at 11.5–12.5. Then, the samples were centrifuged, the supernatant collected, and the pH was lowered to 4–4.5. Similarly to the two-stage process, the samples were purified through dialysis utilizing cellulose membranes, followed by submerging the samples in distilled water with constant renewal for 72 h, freezing the samples at −40 °C for 2 h, and freeze-drying for 48 h.

### 2.3. Characterization Techniques

#### 2.3.1. Yield

The yield of each sample was calculated as the ratio between the final amount obtained and the initial raw material used.

#### 2.3.2. Chemical Composition

Protein quantification: The nitrogen content of the samples was determined in triplicate using the Dumas method with a Flash 2000 N/C Analyser (Thermo Scientific, Waltham, MA, USA). A nitrogen-to-protein conversion factor of 5.71 was used [28].

Lipid quantification: The lipid content was determined in triplicate utilizing the Soxhlet method [29,30], i.e., by condensing heated hexane to the samples to extract the lipids, and then calculating the change in sample weight before and after the treatment.

Ash quantification: Ash content was determined in triplicate by calcining 1 g of sample at 550 °C in a muffle furnace for 4 to 5 h [30], and then calculating the change in sample weight before and after the treatment.

Moisture content: The moisture content was determined in triplicate by heating 0.5–1.0 g of sample in a furnace at 105 °C for 24 h [30], and then calculating the change in sample weight before and after the treatment.

#### 2.3.3. Amino Acid Composition

The amino acid composition of keratin was obtained following the procedure described in Felix et al. [31]. Briefly, keratin protein was dissolved in 6 M hydrochloric acid and incubating it in an oven at 110 °C for 24 h. After hydrolysis, pH was adjusted to 7 using 6 M NaOH and the samples were filtered through a Whatman glass microfiber filter (GF/C). Finally, the samples were diluted (1:500) by adding doubly distilled water. Reverse phase HPLC was carried out with a precolumn fluorescence derivatization with o-phtaldialdehyde (SIL-9A Auto Injector, LC-9A Liquid Chromatograph, Shimadzu Corporation, Kyoto, Japan).

#### 2.3.4. Water Imbibing Capacity (WIC)

**Figure 2:**
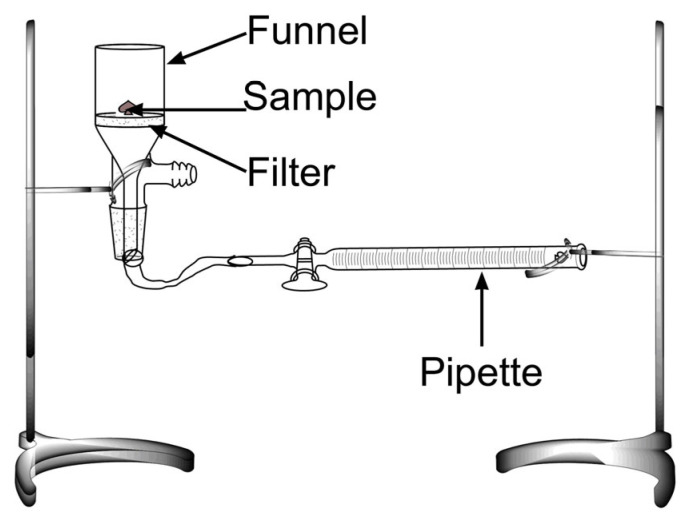
Image of the setup used for the water imbibing capacity tests.

The WIC of the samples was measured utilizing a Baummann apparatus (Figure 2), according to Cuadri et al. [32]. Briefly, a glass Buchner funnel equipped with a borosilicate filter (ROBU Glasfilter-Geraete GmbH, Hattert, Germany) was connected to a horizontal graduated pipette filled with distilled water to the same level as the filter. A 0.5 g thin layer sample was placed on the filter while closing the funnel mouth with an airtight lid. The amount of liquid water absorbed was recorded over time until equilibrium was reached.

Then, the WIC was determined from the change in sample weight initially and when the steady-state was achieved.

#### 2.3.5. Water Absorption Modelling

The average particle size in dry and wet conditions was determined to model the water absorption profiles. The particle size was measured with a laser diffraction size analyzer Mastersizer 2000 (Malvern Panalytical, Malvern, UK), following the ISO 13320:2020 standard. In this way, the particles were dispersed by ultrasound (vibration feed rate 55%) and aspirated (disperser pressure 1 bar) towards the lens, where they hit the 633 nm laser, causing a blockage of 0.5–6% in the lens. The scattering angle of the laser was recorded for 20 s with 20,000 images. The particle size was calculated using Fraunhofer’s theory of light scattering, assuming a model of a sphere equivalent to volume [33]. The results were expressed through the Sauter’s diameter (D [2,3]), which is the surface-weighted mean.

The protein particles’ water uptake kinetics were analyzed using Fick’s second law for a spherical geometry (Equation (1)) [34]. The water concentration profiles during the water uptake were generated with a Matlab code using the Ode15s differential equation solver.

(1) $$\frac{\partial C}{\partial t}=D\left(\frac{2}{r}\frac{\partial C}{\partial r}+\frac{\partial^{2}C}{\partial r^{2}}\right)$$

The swelling geometry was considered by performing the modeling and calculating the diffusivity for both the dry (*D*_d_) and the saturated (wet, *D*_w_) particle diameters (*f*_d_ and *f*_w_, respectively). *f*_w_ was estimated by assuming an additive volume of the material and the liquid and considering the saturated uptake (in ml liquid/g material), which defined the WIC parameter. The density of the different materials was used in the calculations. For each case, the diffusivity was considered a constant, and the average diffusivity for the dry and wet systems was determined. The averaging was made in two ways, based on either the direct values *D*_av1_ = (*D*_d_ + *D*_w_)/2 or the logarithmic values *D*_av2_ = exp((ln(*D*_d_) + ln(*D*_w_))/2).

#### 2.3.6. Solubility and Z-Potential

Aqueous dispersions (ca. 1 g protein/40 mL) were prepared to measure the protein solubility at different pH values, and the pH of aliquots was adjusted to alkaline pH values with a 6 N NaOH solution and acid pH values with a 2 N HCl solution. Samples were homogenized and centrifuged for 20 min at 15,000 rpm and 10 °C. The supernatants were collected for protein content measurement by the Markwell method [35]. Solubility was expressed as a percentage (g soluble protein/100 g isolate in the sample).

The isoelectric point was measured using a Zetasizer 2000 device (Malvern Instruments, Malvern, UK). Therefore, different samples were prepared at 1 wt% at a pH value with buffers. Before analysis, the samples were stirred at 20 °C and centrifuged at 10,000× *g* for 10 min in an RC5C Sorvall centrifuge (Sorvall Instruments, Silver Spring, MD, USA). After that, the samples were measured in triplicate at 20 °C. The zeta potential was calculated using the Henry equation and the Smoluchowski approximation from electrophoretic mobility. The isoelectric point corresponds to the point where the potential value is zero, at which all charges of particles are neutralized [36].

#### 2.3.7. Fourier Transform Infrared Spectroscopy (FTIR)

The FTIR spectra of the samples were obtained using a PerkinElmer Spectrum 100, coupled to an ATR single-reflection Golden Gate unit (Graseby Specac LTD, Kent, UK) and a triglycine sulfate detector. All the spectra were obtained with an average of 16 scans and a scanning resolution of 4.0 cm^−1^. Samples were dried in a desiccator for 1 week before the tests. All the spectra were normalized against the peak at 917 cm^−1^, which is assigned as the skeletal stretching [37].

#### 2.3.8. Size Exclusion High-Pressure Liquid Chromatography (HPLC)

The protein extractability and polymerization in the samples were evaluated by size-exclusion high-performance liquid chromatography (HPLC) using a Waters 2690 HPLC instrument and a Phenomenex BIOSEP SEC-4000 column following methods previously described [13]. This procedure consisted of a three-stage extraction process using a 0.5 wt% SDS and 0.05M NaH_2_PO_4_ solution as the extraction buffer. For each sample, the prepared suspension was agitated at 2000 rpm for 5 min and centrifuged at 12,500 rpm for 30 min, obtaining the soluble extractable from the supernatant (Extraction 1). Subsequently, the resulting pellet from the 1st extraction was resuspended in fresh SDS-phosphate buffer solution, ultrasonicated for 30 s with a Sanyo Soniprep 150 Ultrasonic Disintegrator, and centrifuged for 30 min at 12,500 rpm. Once again, the soluble extractable was obtained from the supernatant (Extraction 2). Finally, the third extraction consisted of ultrasonication for 2 min of the centrifugated pellet with fresh SDS-phosphate solution (Extraction 3). The total protein extraction from the non-functionalized keratin was used as a reference for normalization. All the measurements were carried out in triplicate.

The resulting chromatograms (Supplementary Material Figure S1) used for analysis were obtained by UV-detection using a Waters 996 Photodiode Array Detector at a wavelength of 210 nm. The chromatograms were divided into 1–15 min for the polymeric fraction (named as total PP) and 15–30 min for the monomeric fraction (named as total MP).

#### 2.3.9. Visual Absorption Test (VAT)

Swelling and liquid spreading properties of the dried samples in blood and 0.9 wt% NaCl (saline solution) were assessed utilizing a visual absorption test, as previously described [38]. Briefly, 100 mg of the extracted sample was placed on a glass petri dish, and 100 µL aliquots of the tested liquid (blood or saline solution) were gently pipetted onto the material. A timer was set after the addition of the first aliquot. Each aliquot was allowed to soak before adding the subsequent aliquot, and the addition stopped when liquid saturation was apparent. The VAT was calculated as grams of liquid required to reach saturation per gram of dry powder and reported as the average from duplicates.

### 2.4. Statistical Analysis

Calculated in Excel, mean values and standard deviations have been used to compare samples and their behavior.

## 3. Results

### 3.1. Extraction and Functionalization of Keratin

#### 3.1.1. Product Yield and Chemical Composition

Both the functionalization process and the type and amount of functionalization agent impacted the product yield and the chemical composition of the products (functionalized keratin). Similarly, as in previous studies [39,40], a yield of ca. 35% was obtained for KE (non-functionalized keratin). However, the yield normally depends on the processing conditions (temperature, time, pH, nature, and content), and yield from 30 to 95% has been previously reported [41,42]. Additionally, to produce soluble keratin, reagents such as 2-mercaptoethanol or sodium bisulfite under moderately alkaline conditions have been evaluated to reduce disulfide bonds. However, from an industrial and environmental point of view, substituting those reducing reagents with NaOH, which results in alkaline hydrolysis, is more viable [40]. Functionalizing the KE generally decreased yield to 14–24%, with the highest yield for the 1FED10 and 1FED25 samples (one-stage process).

The chemical composition of the KE samples (Table 1), with a protein content of 68%, an ash content of 12%, and a lipid content below 1%, corresponded well with previously reported keratin results [30]. Functionalization of the samples resulted in a general increase in protein content (to 80–95%), with a simultaneous decrease in ash content (to 0.3–1.9%), with the highest protein content and lowest ash content in 2FS25 and 1FS25 samples (Table 1).

The low ash content in the functionalized samples, and thus a high protein content, is the result of the dialysis step carried out at the end of the functionalization process. The dialysis step aimed to remove impurities and excess reagents, thus eliminating the inorganic residues remaining in the protein. Similar studies on the functionalization of keratin to obtain absorbent capacity are scarce. However, Brown et al. report keratin production from wool with a similar protein, ash, and lipid content for wool fibers after hexane extraction [43] to our functionalized samples. However, different types of keratin might be expected if extracted from wool rather than from chicken feathers. Chicken feathers contain β-keratins, which are part of hard avian and reptilian tissues, such as feathers [44]. The samples’ high protein and low ash content are likely favorable for their functional properties and use as absorbent materials.

#### 3.1.2. Amino Acid Composition

The amino acid composition of the KE sample (Table 2) corresponded well with results from previous investigations [32,33] with a high glycine, serine, and valine content and a cysteine content below 2%. Functionalization of the KE via acylation typically occurs via the condensation of -COOH groups on the lysine (–NH_2_) residues. The presence of a significant amount of arginine, glutamine, and lysine in the samples is thereby critical for more efficient acylation processes, i.e., more condensation of -COOH on the lysine residues. This reaction was previously stated in the literature in which the reaction mechanism of the functionalized protein was included [45].

#### 3.1.3. Swelling Capacity of the Extracted Keratin

**Figure 3:**
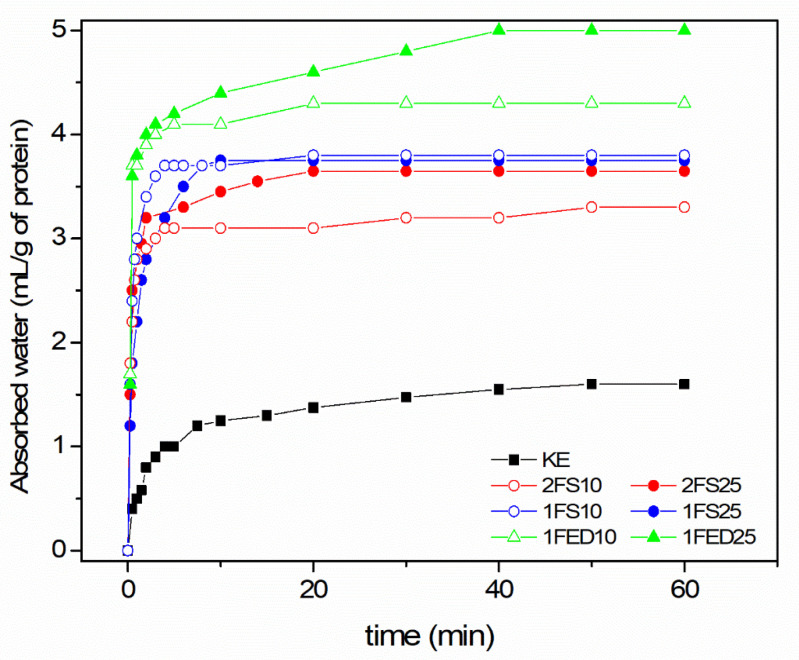
Water Imbibing Capacity of keratin (KE) and sample of functionalized keratin using a two- (2) or one- (1) stage process with either Succinic Acid (SA) or EDTAD (ED) of 10% (10) or 25% (25) as functionalizing agent.

The Water Imbibing Capacity (WIC) increased when the KE was functionalized (Figure 3), with the highest capacity for the 1FED10 and 1FED25 samples (functionalized in one-stage using EDTAD). In these samples, the water absorption was increased three-fold (from 160 to 430 and 500) in relation to the non-functionalized sample (KE; Table 3).

In previous studies [20], functionalizing soy protein isolate through acylation has shown that the WIC is directly connected to the amount of functionalizing agent utilized. However, in the present study on KE, the amount of functionalization agent played a role in the two-stage process using SA as a functionalizing agent, while it did not show to affect the WIC in the one-stage process (Figure 3). It was possible to reach ca. 30% more WIC of the keratin if the SA content was increased to 25 wt% using the two-stage process. On the contrary, in the one-stage process using EDTAD as a functionalizing agent, more WIC was obtained in the keratin when 25 wt% EDTAD was used (Figure 3). Thus, the impact of the amount of functionalization agent added to a protein seemed to be dependent on the nature of the reagent used and the functionalization/extraction route.

**Figure 4:**
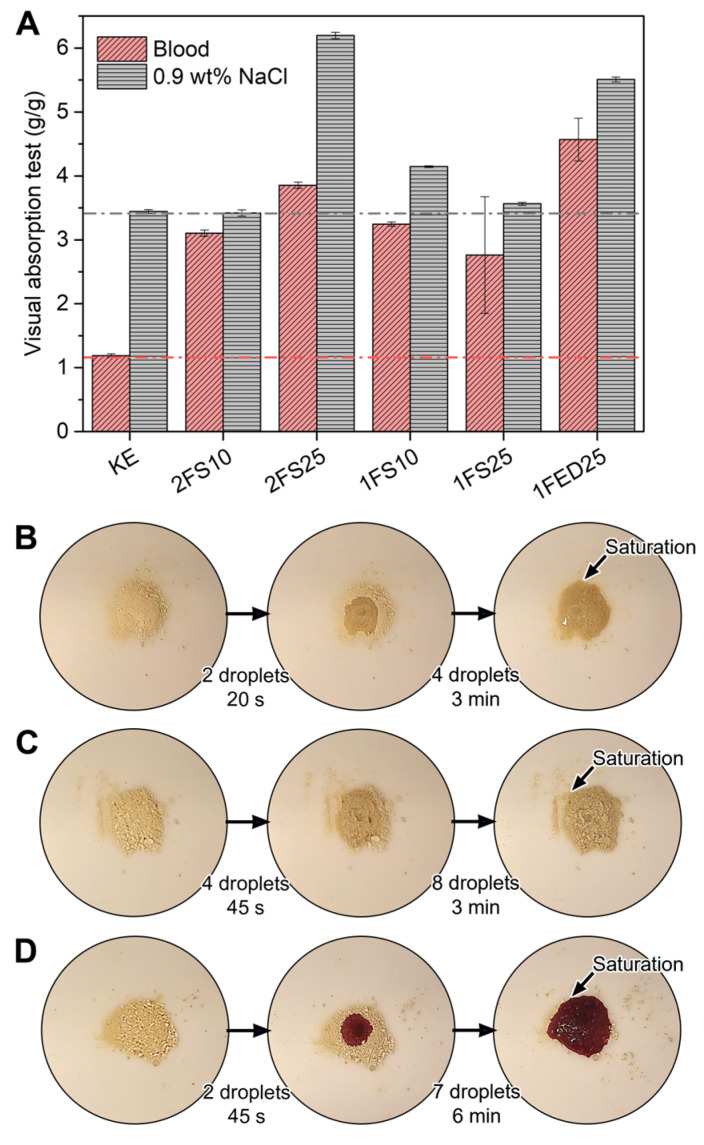
Visual Absorption Test for the samples utilizing blood and saline solution (**A**): the addition of saline until the saturation point for keratin (**B**) and keratin two-stage functionalized with 25 wt% SA (**C**), and of blood for keratin one-stage functionalized with 25 wt% EDTAD (**D**).

An absorbent material is used in many applications, where medical, hygiene, and daily care products absorbing liquids such as urine and blood, have a large share [15]. The functionalized KE evaluated here showed a high potential to absorb such liquids (Figure 4A) as visualized by an absorption test (VAT) utilizing blood and a saline solution (0.9 wt% NaCl). The greatest saline absorption (ca. 6 g/g) was displayed by the 2FS25 samples, while the highest blood absorption (4.5 g/g) was obtained for 1FED25. Thus, KE, 2FS25, and 1FED25 were selected to visualize the liquid spreading properties (Figure 4B–D, respectively). The results indicated similarities for saline spreading in KE (Figure 4B) and 2FS25 (Figure 4C) despite the highly different VAT values (Figure 4A). The saline spreading was rapid throughout the dry powder for all samples, shown for most representative samples (KE, 2FS25, and 1FED25) in Supplementary Material (Video S1).

Although the 1FED25 sample had the highest blood absorption, with an increase of 350% compared to KE (Figure 4A), the second-highest blood absorption was found for the 2FS25 sample. As SA is known for being less expensive and contributing less greenhouse gas emissions to the environment than EDTAD [36,37,38], these findings point towards the potential of SA to produce KE functionalized samples with increased liquid swelling properties compared to EDTAD. The functionalized samples’ improved interaction and blood swelling properties compared to KE are shown as representative samples (KE, 2FS25, 1FED25) in Supplementary Video S2. Contrary to the saline VAT results, the blood VAT revealed that all functionalized materials (SA and EDTAD) absorbed the blood only on the spot where the droplet touched the powder and had a slow liquid spreading, taking up to 6 min to reach saturation (Figure 4D). Additionally, when the blood-saturated functionalized materials were stirred, non-colored parts of the sample were spotted, indicating that not all the powder participated in the swelling (Supplementary Material Video S2). These results suggest that the functionalized materials presented a gel-blocking effect, a common defect in absorbent materials when too rapid swelling of the particles occurs, which has been shown in previous studies to hinder the liquid from spreading evenly through the powder [25]. Improvements in saline swelling and a more rapid blood uptake are interesting properties to focus on for KE-based absorbent materials to be used in sanitary products such as incontinence and menstruation pads.

#### 3.1.4. Water Uptake Kinetics

**Figure 5:**
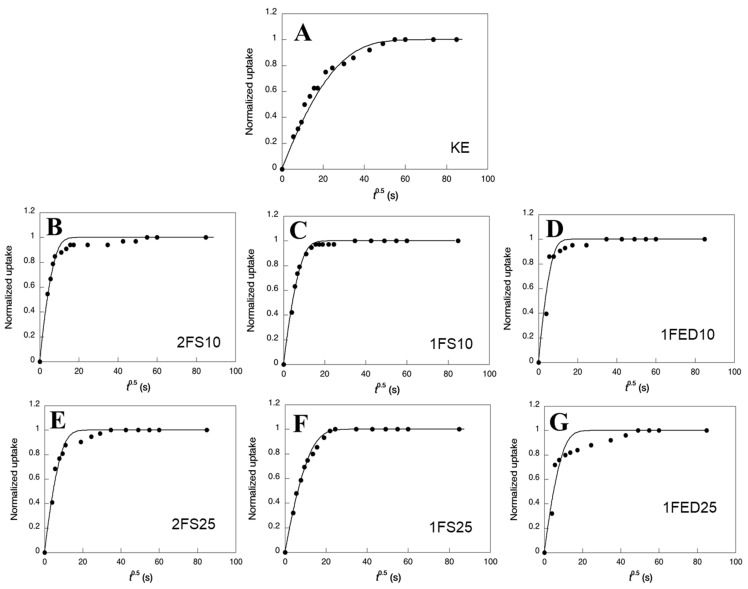
Normalized water uptake as a function of the square root of time (t^0.5^) for the different systems obtained: (**A**) KE, (**B**) 2FS10, (**C**) 1FS10, (**D**) 1FED10, (**E**) 2FS25, (**F**) 1FS25, and (**G**) 1FED25. Data points refer to experimental values and the curves refer to the modeling.

Modeled and experimental uptake curves showed a two-step uptake for essentially all samples (Figure 5), although this was most apparent in the 1FED samples and least evident in the 1FS samples.

The two-step feature was also stronger with increasing content (25% compared to 10%) of the functionalizing agent. The diffusivity data showed that the KE samples had the lowest average water diffusivities (Table 4); hence, functionalization increased the uptake rate. The consequence of a strong two-step uptake was that the final uptake often occurred after significantly longer periods of time than the first uptake (Figure 5). The second-step uptake was, hence, quite slow. Considering the whole uptake and the average diffusivities, the EDTAD functionalized samples showed the largest water diffusivity (Table 4). Corresponding with that, those samples also had the highest WIC values as shown above (Figure 3). The low diffusivity could result from a mass transfer limitation due to strong interactions between the liquid molecules and the protein matrix active sites. The result agrees with previous work demonstrating the low diffusivity of rGO and Fe_3_O_4_ particles immobilized on enzymatic systems [46].

#### 3.1.5. Changes in Secondary Structure by the Functionalization of the Keratin

**Figure 6:**
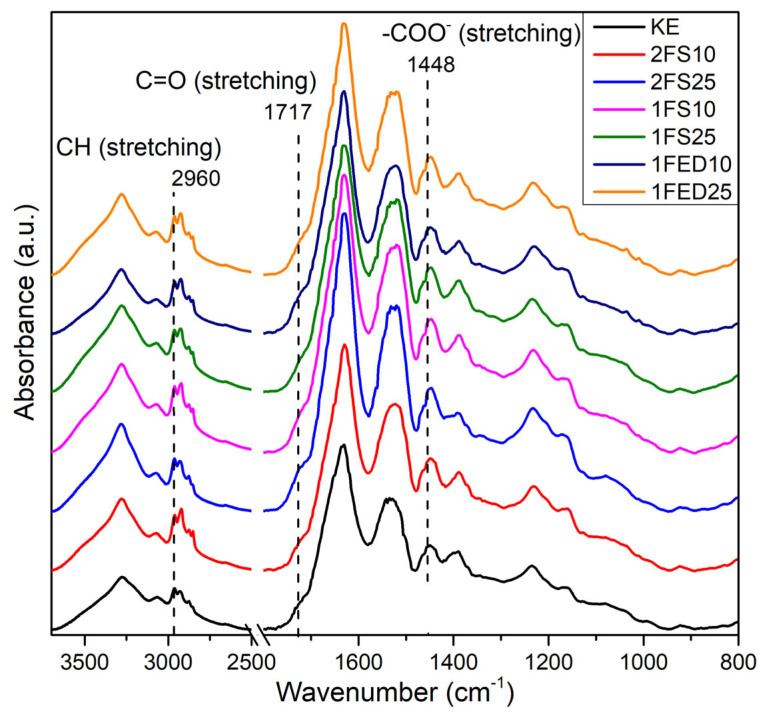
FTIR profiles of keratin (KE) and keratin functionalized in one (1)- or two (2)- stages, with SA (S) or EDTAD (ED) at 10% and 25%.

The FTIR of the KE samples shown in Figure 6 reveals a standard spectrum for proteins, i.e., with the characteristic amide I (1632 cm^−1^) and amide II (1519 cm^−1^) peaks corresponding to C=O and CN (stretching), and NH (bending) coming from the peptide bond from the keratin, respectively [47]. The small band in the range 1300–1200 cm^−1^ in Figure 6 is the amide III band that results from C–N stretching and N–H bending [10]. The structural conformation of keratin is mainly composed of α-helix and β-sheets [48], which agrees with the shape of the amide I and III peaks.

KE and all the functionalized samples had a broad and strong OH stretching region (3500–3100 cm^−1^), with the lowest/smaller O–H region observed for KE, 2FS10, and 1FED10 (Figure 6). In addition, all functionalized samples (independent of process, agent, or content) resulted in sharp and strong bands between 2960–2875, ascribed to an increase in symmetric and asymmetric stretching of CH bonds [49]. The increase in the CH bonds is possibly a consequence of the SA and EDTAD being chemically bonded and grafted to the protein. The carbonyl stretching bands at 1717 and 1448 cm^−1^ were assigned to the carboxylic acid and carboxylate groups, respectively [50]. The carbonyl stretching increased in the treated samples, with the strongest for 2FS25 and 1FS10. The result is again ascribed to functionalizing the protein with SA and EDTAD, forming pendant carboxylic acid groups [25]. A summary of the peak assignments is provided in Table S1.

The increase in OH and carbonyl-related signals in Figure 6 indicates a successful keratin functionalization using SA and EDTAD. Similarly, as has been shown in previous studies [17], stronger signals were obtained for the samples treated at the highest concentration of the agents herein used (1FS25, 2FS25, 1FED25) as compared to the low concentration samples (1FS10, 2FS10, 1FED10). The increase in carboxylic acid content also correlated with the high swelling results obtained for these samples (see Figure 3 and Figure 4). The results agree with previous work reporting the functionalization of lysine moieties in proteins via acylation with EDTAD and SA. The acylation agents are grafted to the protein via nucleophilic attack, leaving one or three carboxylate (–COO^−^) pendant groups if SA or EDTAD are used, respectively [27].

#### 3.1.6. Solubility and Z-Potential

**Figure 7:**
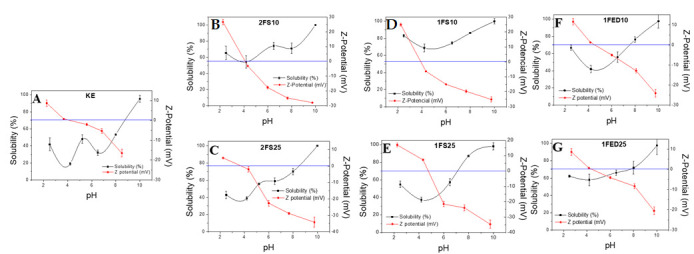
Solubility and Z-potential of the different keratin proteins extracted and functionalized: (**A**) keratin extracted; (**B**,**C**) keratin extracted and functionalized with succinic anhydride at 10% and 25% in two stages; (**D**,**E**) keratin extracted and functionalized with succinic anhydride at 10% and 25% in one stage; (**F**,**G**) keratin functionalized with EDTAD at 10% and 25% in one stage.

Figure 7 shows the solubility curve and the Z-Potential obtained for the extracted keratin (used as reference) and the functionalized keratin systems, either with SA (Figure 7B–E) or EDTAD (Figure 7F,G), as a function of pH. The highest solubility values were obtained at basic pH for all the samples, while the lowest solubility was at pH 4–5, which corresponded well with the pH value of which the Z-Potential was 0, indicating the isoelectric point of the sample. Thus, our findings corresponded well with results from previous studies, reporting an isoelectric point at pH 4–5 for keratin [51]. The functionalized samples evaluated here generally showed a higher solubility than KE samples, demonstrating an improved interaction with water obtained by the functionalization. In general, the functionalized samples showed a low solubility in the pH range of 3–6, which can be seen as beneficial, as this pH range corresponds to that of body fluids. Thereby, material leakage should be expected to be low if used in sanitary applications.

#### 3.1.7. Protein Crosslinking

**Figure 8:**
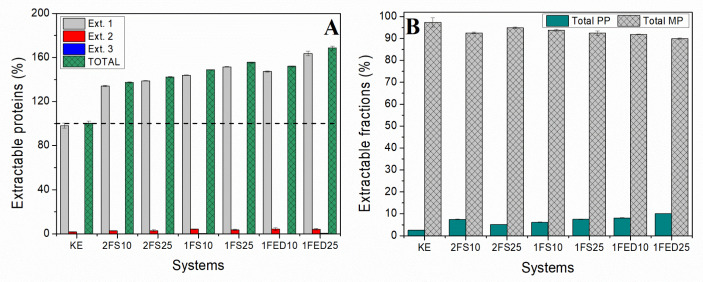
(**A**) Extractable proteins from serial extractions and (**B**) total polymeric (PP) and monomeric (MP) extractable fractions from HPLC and normalized to the KE sample for keratin (KE) and keratin functionalized in one (1)- or two (2)- stages, with SA (S) or EDTAD (ED) at 10% and 25%.

All the functionalized samples showed a higher level of extracted protein as compared to the KE samples when mirroring the total protein content of the samples (Figure 8A).

As the protein extraction was low at the second extraction and near to zero at the third extraction (Figure S1), most of the protein content in the samples was extracted using an SDS buffer and sonication in sequential steps. The highest amount, and close to all the soluble proteins, were extracted during the first extraction, indicating that protein extractability occurred mainly by disrupting secondary bonds [13]. The fact that a significantly higher protein extractability was obtained for samples functionalized with 25% EDTAD (1FED25) as compared to samples functionalized with 25% succinic anhydride (2FS25 and 1FS25) might be the result of conformational changes of the proteins resulting in differences in light uptake/scattering of the samples as has been described in previous studies [13]. Such a difference in conformational changes might result from inserting three negatively charged COO^−^ groups per EDTAD molecule compared to the single carboxyl group included when using succinic anhydride. The low cysteine content (Table 2) in KE might explain the low level of disulfide bonds in the samples, as all proteins are extracted after breaking the secondary bonds.

Protein extractions followed by SE-HPLC revealed that more than 90% of the protein was monomeric in all samples (Figure 8B). However, the KE samples showed the lowest amount of polymeric protein (Total PP), indicating a higher degree of polymerization of the KE proteins with functionalization. Capezza et al. (2020) have suggested that increased hydrodynamic volume by inserting COO– negatively charged groups could induce steric hindrance in the polymer chains [52]. However, the functionalization using either SA or EDTAD is also known to result in an increased crosslinking and formation of polymer fractions in line with the results presented here [46]. The increase in pendant hydrophilic groups can also increase electrostatic repulsion between the protein chains, thereby decreasing their cohesion when in contact with water and promoting more protein extractability/solubility (Figure 8) [27].

## 4. Conclusions

Keratin is available in large quantities in poultry feathers, which is a substantial side-stream from the poultry industry. Functionalization of keratin results in highly improved absorption properties of water, saline, and blood, increasing the product’s value with potential uses in various applications where absorption properties are required. The process was compared between the extraction, drying, and functionalization of the keratin (two-stages) and the extraction with subsequent functionalization of the proteins (one-stage). It was demonstrated that the extraction of the keratin with the in situ functionalization of the keratin results in the highest liquid absorption capacity, providing a simplified and less energy-demanding production method. The functionalization with 25 wt% of EDTAD as a functionalization agent resulted in higher absorption of water, saline, and blood than other combinations evaluated in this study. Overall, the benefits of a one-stage as compared to a two-stage process, is savings of energy and other resources, thereby resulting in a more environmentally friendly process.

## Figures and Tables

**Table 1:** Summary yield and composition of the different samples after functionalization with succinic anhydride (SA) or EDTAD at either 10 or 25 wt% of acylation agent content.

Samples	Extraction–Functionalization		Composition		
	Funct. Agent—Content	Yield	Protein (%)	Ash (%)	Lipids (%)
KE	-	34.87%	67.77 ± 1.30	11.80 ± 0.28	0.88 ± 0.25
2FS10	SA—10%	17.38%	79.40 ± 0.29	0.38 ± 0.01	1.80 ± 0.03
2FS25	SA—25%	13.98%	91.79 ± 0.72	1.88 ± 1.51	0.99 ± 0.62
1FS10	SA—10%	20.94%	78.40 ± 2.55	0.30 ± 0.18	2.36 ± 0.18
1FS25	SA—25%	20.33%	94.75 ± 0.68	0.53 ± 0.16	0.93 ± 0.79
1FED10	EDTAD—10%	23.85%	80.05 ± 1.51	0.42 ± 0.09	0.74 ± 0.02
1FED25	EDTAD—25%	23.21%	84.80 ± 1.18	0.42 ± 0.01	0.82 ± 0.47

KE = keratin, sample names (2FS10-1FED25) refer to stages of process (2 or 1), functionalization agent (S = SA, ED = EDTAD), and concentration (10 or 25%).

**Table 2:** Amino acid composition of keratin protein.

Amino Acid	Content (g/100 g Protein)
Asp	6.2
Thr	4.0
Ser	10.9
Gln	11.6
Gly	9.5
Ala	4.4
Cys	1.8
Val	10.4
Met	0.3
Ile	5.1
Leu	8.5
Tyr	1.8
Phe	5.0
His	0.3
Lys	1.9
Arg	7.0

**Table 3:** Water absorption of the different systems obtained.

Systems	Water Absorption (%)
KE	160 ± 5
2FS10	330 ± 21
2FS25	370 ± 35
1FS10	380 ± 14
1FS25	380 ± 28
1FED10	430 ± 17
1FED25	500 ± 42

**Table 4:** Parameters used for the water swelling kinetics modeling.

Systems	*f*_d_ (µm)	*D*_d_ (cm^2^/s)	*f*_w_ (µm)	*D*_w_ (cm^2^/s)	*D*_av1_ (cm^2^/s)	*D*_av2_ (cm^2^/s)	*f*_w_/*f*_d_
KE	1.6 ± 1.2	1 × 10^−12^	2.0 ± 0.01	1 × 10^−12^	1 × 10^−12^	1 × 10^−12^	1.2
2FS10	2.8 ± 1.5	4 × 10^−11^	4.2 ± 0.03	9 × 10^−11^	7 × 10^−11^	6 × 10^−11^	1.5
2FS25	4.0 ± 2.1	6 × 10^−11^	6.15 ± 0.15	1 × 10^−10^	1 × 10^−10^	9 × 10^−11^	1.5
1FS10	16 ± 3	1 × 10^−9^	25 ± 0.2	3 × 10^−9^	2 × 10^−9^	2 × 10^−9^	1.6
1FS25	2.0 ± 0.8	8 × 10^−12^	3.2 ± 0.04	2 × 10^−11^	1 × 10^−11^	1 × 10^−11^	1.5
1FED10	25 ± 5	4 × 10^−9^	42 ± 0.5	1 × 10^−8^	8 × 10^−9^	7 × 10^−9^	1.7
1FED25	43 ± 6	6 × 10^−9^	77 ± 1	2 × 10^−8^	1 × 10^−8^	1 × 10^−8^	1.8

## Data Availability

The raw/processed data required to reproduce these findings cannot be shared at this time as the data also forms part of an ongoing study.

## Supplementary Materials

The following supporting information can be downloaded at https://www.mdpi.com/article/10.3390/polym15020351/s1, Figure S1: Resulting chromatograms from the HPLC analyses of the different samples: (A) KE, (B) 2FS10, (C) 2FS25, (D) 1FS10, (E) 1FS25, (F) 1FED10, and (G) 1FED25. The curves on each spectrum represent the different extractions, with the biggest shown being the first extraction. The values on the spectra represent the time intervals for separating the different molecular weight fraction; Table S1: FTIR assignments of the different systems obtained: KE (keratin extracted), 2FS10, and 2FS25 (keratin extracted and functionalized with succinic anhydride at 10% and 25% in two stages), 1FS10 and 1FS25 (keratin extracted and functionalized with succinic anhydride at 10% and 25% in one stage), 1FED10 and 1FED25 (keratin functionalized with EDTAD at 10% and 25% in one stage); Video S1: Representation of saline spread, shown for most representative samples (KE, 2FS25 and 1FED25), Video S2: Improved interaction of representative samples (KE, 2FS25, 1FED25) and blood swelling properties, compared to KE.
