# Supplementary material for: Functionalization Routes for Keratin from Poultry Industry Side-Streams—Towards Bio-Based Absorbent Polymers
Source: Polymers (Basel). 2023 Jan 9;15(2):351. doi: 10.3390/polym15020351 (PMC9863878; doi:10.3390/polym15020351)
Supplement: Supplementary file 1 [file polymers-15-00351-s001.zip › polymers-2135212-supplementary.pdf]

Functionalization routes for keratin from poultry industry side-streams – towards bio-based absorbent polymers

SUPPLEMENTARY MATERIAL

Table S1. FTIR assignments of the different systems obtained: KE (Keratin extracted), 2FS10 and 2FS25 (Keratin extracted and functionalized with Succinic Anhydride at 10% and 25% in two stages), 1FS10 and 1FS25 (Keratin extracted and functionalized with Succinic Anhydride at 10% and 25% in one stage), 1FED10 and 1FED25 (Keratin functionalized with EDTAD at 10% and 25% in one stage).

Band (cm^{-1})	Assignment
1632	C=O and CN (stretching)
1519	NH (bending)
3500-3100	OH (stretch, H bonded)
2960-2875	CH (stretching)
1717	Carbonyl (stretching)
1448	Carbonyl stretching vibrations from carboxylate (-COO-)

Figure S1. Resulting chromatograms from the HPLC analyses of the different samples: (A) KE, (B) 2FS10, (C) 2FS25, (D) 1FS10, (E) 1FS25, (F) 1FED10, (G) 1FED25. The curves on each spectrum represents the different extractions, with the biggest shown being the first extraction. The values on the spectra represent the time intervals for separating the different molecular weight fraction.

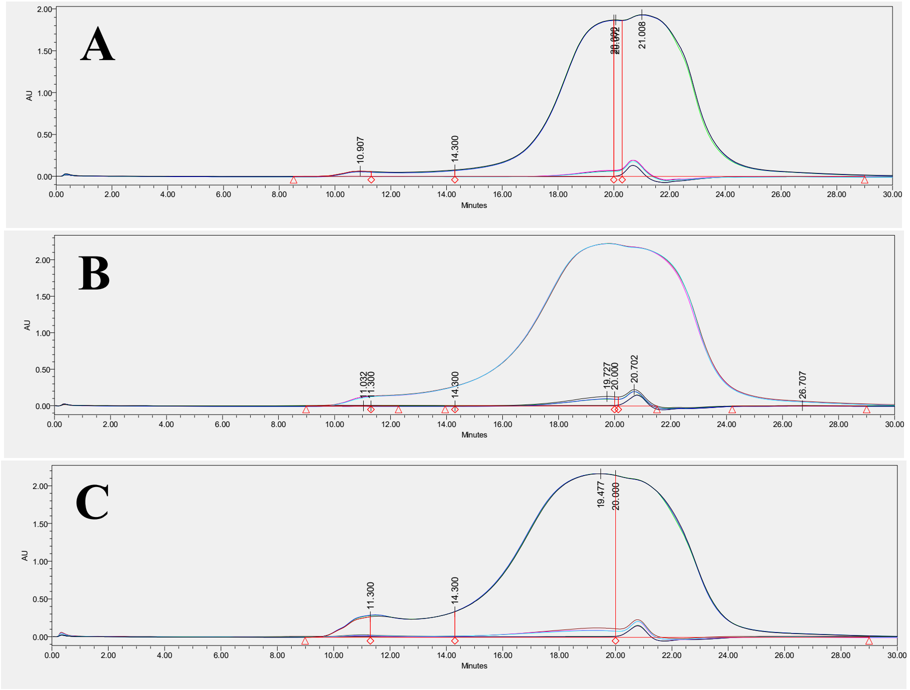

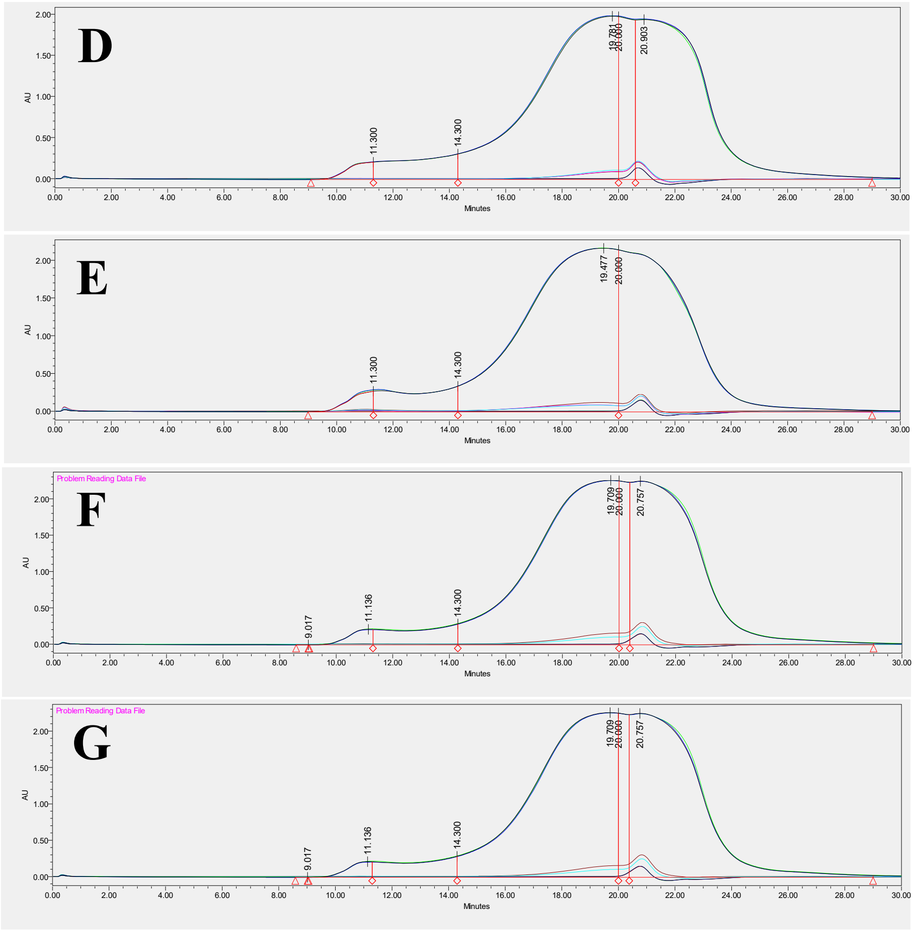

D
E
F
G
Problem Reading Data File
AU
Minutes
